# Self‐induced incipient ‘eclogitization’ of metagranitoids at closed‐system conditions

**DOI:** 10.1111/jmg.12665

**Published:** 2022-05-17

**Authors:** Simon Schorn

**Affiliations:** ^1^ Department of Petrology and Geochemistry, NAWI Graz Geocenter University of Graz Graz Austria

**Keywords:** calculated chemical potential relationships, eclogitization, high‐pressure metamorphism, granitoids

## Abstract

The incipient development of diagnostic high‐pressure assemblages—the ‘eclogitization’—of granitoids, such as plagioclase breakdown and small‐scale formation of garnet and phengite does not require exogenous hydration because unlike dry protoliths like basalt/gabbro or granulite, granitoids s.l. contain crystallographically bound H_2_O in biotite. During high‐pressure overprint, partial biotite breakdown causes a localized increase in the chemical potential of H_2_O (*μ*H_2_O). Transport of H_2_O into nearby plagioclase induces the formation of diagnostic eclogite facies assemblages of jadeite–zoisite–K‐feldspar–quartz ± kyanite ± phengite that pervasively replace former cm‐sized plagioclase without requiring the participation of free H_2_O. Depending on pressure–temperature evolution, similar textures may involve albite instead of jadeite, consistent with the general absence of Na‐clinopyroxene in high‐pressure metagranitoids and kindred gneisses. Plagioclase breakdown may also occur due to simple burial because compression leads to an increase of *μ*H_2_O, without requiring additional influx of H_2_O at the texture scale. However, the addition of biotite‐derived H_2_O into plagioclase sites likely increases reaction rates. In parallel, ∼100‐*μ*m‐thick complementary coronae involving garnet | phengite–quartz develop at former biotite–plagioclase/K‐feldspar interfaces due to the coupled diffusion of FeO–MgO–H_2_O from biotite towards feldspars and minor CaO in the opposite direction. The reaction textures likely create structural weaknesses and preferential fluid pathways that facilitate further hydration and/or deformation along the prograde path, thereby obliterating the textures. If exogenous H_2_O is introduced, it is accommodated in phengite growing at the expense of igneous K‐feldspar and possibly in epidote‐group minerals. Upon decompression, such hydrated rocks would dehydrate, thereby favouring fluid‐assisted retrogression and loss of diagnostic eclogite facies assemblages at lower pressure. Whereas the prograde reaction textures are only preserved at closed‐system conditions and in the absence of deformation, they are suggested to commonly form during orogenic metamorphism of granitoids and quartzofeldspathic gneisses that dominate the continental crust in high‐pressure terranes such as the Western Italian Alps and the Western Gneiss Region (Norway).

## 1 INTRODUCTION

Metagranitoids and quartzofeldspathic gneisses constitute the bulk of the continental crust involved in orogenic (ultra)high‐pressure (UHP) metamorphism, for example, in the Dabie Shan (China; Carswell et al., 2000), the Tso Morari Massif (India; Palin et al., 2017), or the Western Gneiss Region (WGR) of Norway (Young & Kylander‐Clark, 2015). However, in such high‐pressure terranes, they are rarely the focus of study compared to metamafic lithologies such as eclogites. This is because metagranitoids usually either do not develop eclogite facies assemblages because of impaired equilibration at fluid‐deficient conditions (e.g., Palin et al., 2017; Young & Kylander‐Clark, 2015) or fail to preserve them due to subsequent low‐pressure retrogression driven by dehydration (Proyer, 2003).

Metagranitoids require external hydration in order to fully convert to their eclogite facies equivalents (Proyer, 2003), in particular to form phengitic white mica at the expense of potassic feldspar. As such, H_2_O not only exerts a fundamental catalytic role (Milke et al., 2013; Rubie, 1986) but is also a key chemical component involved in the ‘eclogitization’ of granitoids—their transformation into garnet–Na‐clinopyroxene–phengite rocks. Unlike dry basalt/gabbro or granulite, however, granitoids commonly host minor amounts of structurally bound H_2_O in micas and/or amphiboles. During burial and heating, the breakdown of these minerals may drive local equilibration so long H_2_O is liberated, but because hydrous higher pressure phases (e.g., phengite, paragonite, and/or epidote‐group minerals) form at the expense of igneous assemblages, there is no net production of H_2_O and the ‘self‐induced’ equilibration is limited to the sites and duration of dehydration. Indeed, save for H_2_O addition via fluid infiltration along the prograde path (Tursi et al., 2021), metagranitoids rarely record conditions beyond the high‐pressure amphibolite facies (Young & Kylander‐Clark, 2015). In addition to equilibration triggered by fluid infiltration (e.g., Austrheim, 1987; Schorn & Diener, 2017, and references therein), deformation is a critical factor controlling the degree of metamorphic reaction progress, in particular under high‐pressure–low‐temperature conditions (Gosso et al., 2010, Hobbs et al., 2010, and references therein).

However, high‐pressure equilibration under both closed‐system (i.e., fluid‐absent) and static conditions is not entirely absent. Here the focus lies on low‐strain domains of eclogite facies metagranitoids, where the igneous parageneses are largely preserved. The original assemblages are partly overprinted by various reaction textures during high‐pressure metamorphism, triggered by the incomplete breakdown of igneous biotite. As such, these domains represent snapshots of incipient ‘eclogitization’ of granitoids under static, closed‐system conditions. Published examples of coronitic metagranites of the Italian Western Alps and the Iberian Massif of northern Spain are investigated here using calculated chemical potential relationships and pseudosection modelling to suggest that self‐induced small‐scale equilibration might be a common—albeit rarely preserved—phenomenon in the continental crust affected by high‐pressure metamorphism.

## 2 GEOLOGICAL BACKGROUND

### 2.1 Western Alps

#### 2.1.1 Sesia–Lanzo Zone (Monte Mucrone)

The Sesia–Lanzo Zone (SLZ) is a composite continental unit in the Western Alps (northwest Italy) that consists of polymetamorphic metasediments, mafic and ultramafic rocks, marbles, (meta‐)granites, and (meta‐)granodiorites (e.g., Compagnoni & Dal Piaz, 1977; Oberhänsli et al., 1985). The SLZ is a sliver of Variscan crust that was affected by early‐Alpine (or Eo‐Alpine; Oberhänsli et al., 1985) high‐pressure–low‐temperature metamorphism in the Late Cretaceous (c. 70–65 Ma; Duchêne et al., 1997; Rubatto et al., 1999). It tracks a *P*–*T* path dominated by compression/decompression, leading from prograde blueschist‐ to eclogite facies conditions followed by a greenschist‐grade overprint (e.g., Roda et al., 2012; Regis et al., 2014; Zucali et al., 2020, and references therein). Estimated *P*–*T* conditions range between  500°C and 625°C and 13–25 kbar for the Eo‐Alpine high‐pressure event (see Roda et al., 2012, for a compilation), occurring between 90 and 65 Ma in the SLZ (Oberhänsli et al., 1985; Regis et al., 2014). The investigated Monte Mucrone rocks consist of a suite of metamorphosed granites, granodiorites, and minor quartz‐diorites with *I*‐type character (Oberhänsli et al., 1985) and a Permian intrusion age of 286 ± 2 Ma (Paquette et al., 1989).

The metagranitoids form decametre‐ to kilometre‐sized bodies with medium to coarse grain size, spanning from undeformed to mylonitic domains (Compagnoni & Maffeo, 1973; Früh‐Green, 1994; Koons et al., 1987; Zucali et al., 2020). A strong link between deformation and metamorphic reaction progress is documented for the Mucrone area (Früh‐Green, 1994; Koons et al., 1987), with >85% of the rock volume preserving fabrics, assemblages, and/or reaction textures from the high‐pressure overprint (Zucali et al., 2020). Where fluid infiltration was efficient, the rocks host blueschist to eclogite facies assemblages (Zertani et al., 2021), whereas magmatic parageneses and textures are largely preserved in low‐strain domains (e.g., Bruno & Rubbo, 2006; Oberhänsli et al., 1985; Rubbo et al., 1999). Eo‐Alpine conditions 500°C to 600°C and >16 kbar (Compagnoni & Maffeo, 1973; Oberhänsli et al., 1985; Rubbo et al., 1999) and a clockwise *P*–*T* path are reported for the metagranitoids (Bruno & Rubbo, 2006).

#### 2.1.2 Dora Maira Massif (Brossasco–Isasca Unit)

The Brossasco–Isasca Unit (BIU) of the southern Dora Maira Massif consists of a continental Variscan amphibolite facies basement intruded by granitoids in the Permian (c. 275 Ma; Gebauer et al., 1997, and references therein) overprinted by Alpine high‐pressure metamorphism. Retrogressed orthogneisses and metagranitoids, minor coesite‐bearing pyrope–kyanite whiteschists, kyanite eclogites, and marbles are mapped in the BIU (e.g., Chopin et al., 1991; Chopin & Schertl, 1999; Schertl et al., 1991). Clockwise Alpine high‐pressure metamorphism reached peak pressure conditions of ∼40–43 kbar and 730°C (e.g., Ferrando et al., 2009) at about 35 Ma (Rubatto & Hermann, 2001). Rapid exhumation (20–24 km/Ma; Gebauer et al., 1997) is interpreted to have occurred with fast cooling rates (85°C to 100°C/Ma; Gebauer et al., 1997), driven by buoyancy and faulting (Rubatto & Hermann, 2001).

The studied Brossasco metagranitoid occurs as lenses within orthogneisses in the BIU and represents undeformed portions of the Permian intrusives. It is massive, with porphyritic portions truncated by ductile shear zones and aplitic and pegmatitic dykes (Biino & Compagnoni, 1992). The igneous textures are largely preserved, and magmatic assemblages partly replaced by metamorphic minerals during Alpine high‐pressure metamorphism, estimated at ∼24 kbar and 650°C for a sample of coronitic metagranodiorite (Bruno et al., 2001).

### 2.2 Iberian Massif (Malpica–Tuy Unit)

The Iberian Massif of northwestern Spain is part of the European Variscan orogen (e.g., Fernández et al., 2012, and references therein). The Malpica–Tuy Unit (MTU) consists of metamorphosed lower continental crust that is part of an allochtonous complex in the westernmost portion of the Iberian Massif (e.g., López‐Carmona et al., 2013). The MTU is dominated by felsic metavolcanics, orthogneisses, metagranitoids, and subordinate metasediments, amphibolites, eclogites, and peralkaline gneisses (Ortega & Ibarguchi, 1983). The dominant orthogneisses and minor eclogites record respective peak pressures of ∼13.5 kbar at 625°C and 22.5 kbar at 540°C (Li & Massonne, 2016). The authors interpret the different conditions in terms of a contrasting evolution, with incorporation of the eclogites within the gneisses following exhumation from greater depth. U–Pb dating of eclogitic zircon yields an age of 372 ± 2 Ma for high‐pressure metamorphism (Abati et al., 2010).

Ibarguchi (1995) investigates coronitic metagranitoids from a suite of granitic–granodioritic orthogneisses embedded within felsic gneisses of the MTU. Whereas most of the outcrops display pervasive foliation, low‐strain domains partly preserve the original magmatic assemblage and show an incomplete metamorphic overprint, constrained at 630°C ± 40°C and a minimum pressure of 16.5 ± 1 kbar by the same author.

## 3 REACTION TEXTURES

In all investigated cases, the original magmatic assemblage is biotite, K‐feldspar, plagioclase, quartz, and accessories (zircon, apatite, allanite ± tourmaline, ilmenite, magnetite, and monazite; Biino & Compagnoni, 1992; Bruno et al., 2001; Bruno & Rubbo, 2006; Compagnoni & Maffeo, 1973; Ibarguchi, 1995; Oberhänsli et al., 1985; Rubbo et al., 1999; Tropper & Essene, 1999). Together with igneous textures, the precursor assemblages partly survived high‐pressure metamorphism in domains that have escaped significant hydration and/or deformation. The metamorphic reaction textures are limited to pseudomorphic replacement and the growth of coronae which are similar among the samples, despite their contrasting *P*–*T*–*t* evolution.

### 3.1 Monte Mucrone

#### 3.1.1 Former plagioclase site

**FIGURE 1:**
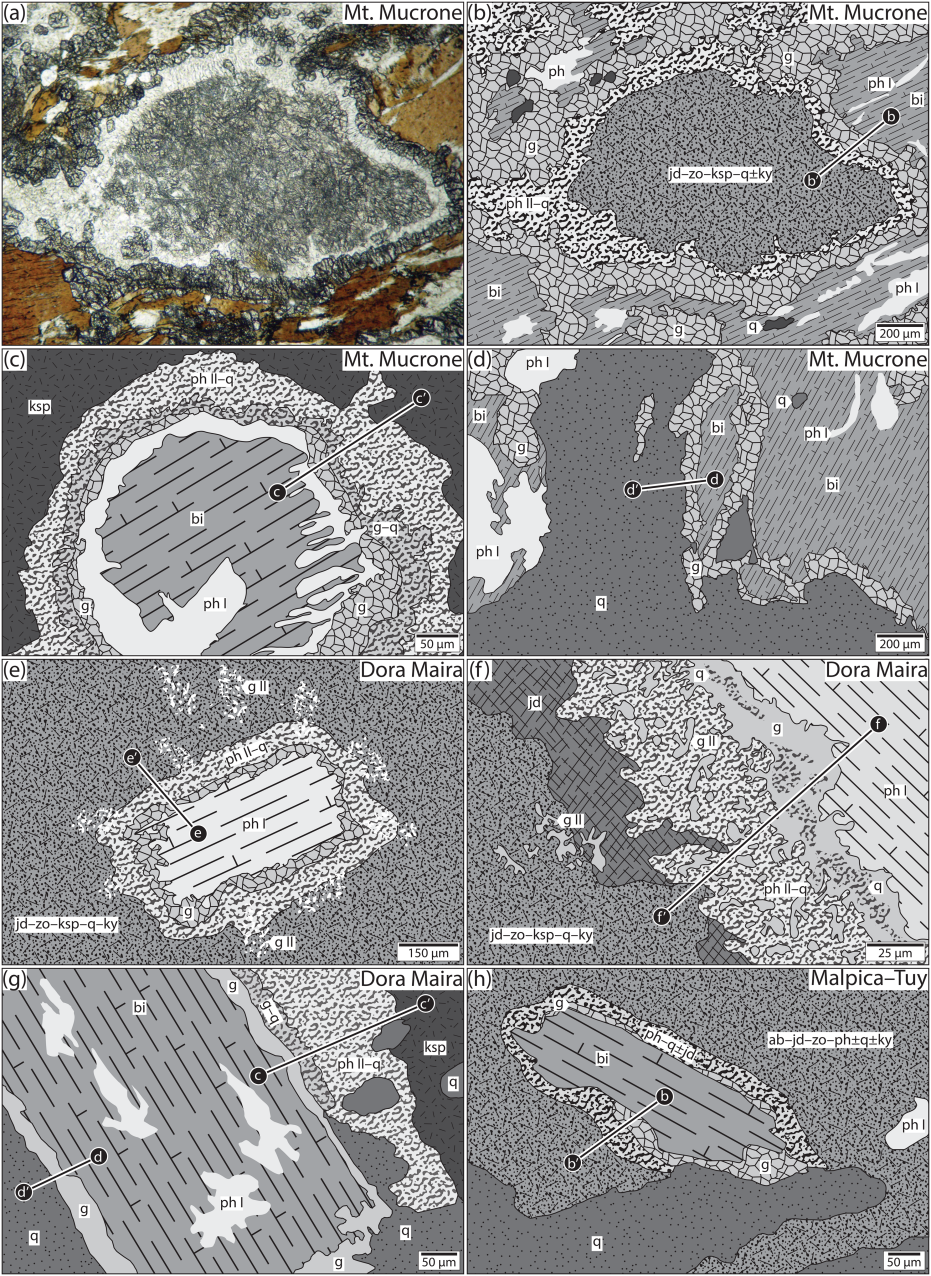
Reaction textures in metagranitoids from (a–g) the Western Alps and (h) the Iberian Massif. Microphotograph (a) is from https://www.alexstrekeisen.it/english/meta/jadeitegranite.php. (c) is redrawn after Rubbo et al. (1999, figure 4). (d) is redrawn from the same source as (b). (e) is simplified from Biino & Compagnoni (1992, figure 7). (f,g) are redrawn from Bruno et al. (2001, figures 3b and 2a, respectively), (h) is from Ibarguchi (1995, figure 3d). Profiles refer to figures showing the same texture

Igneous plagioclase is completely pseudomorphed by a randomly oriented, fine‐grained mesh of jadeite–zoisite–K‐feldspar–quartz ± kyanite forming mm‐sized aggregates (Figure 1a,b). Some pseudomorphs may lack kyanite (Rubbo et al., 1999), whereas others may additionally host muscovite (Tropper & Essene, 1999). Na‐clinopyroxene forms anhedral,  *μ*m‐sized grains within the pseudomorphs. It is nearly pure jadeite (Oberhänsli et al., 1985); Tropper and Essene (1999) report 80–97 and 40–50 mol.% NaAlSi_2_O_6_ for early and more recrystallized clinopyroxene, respectively. Mn, Cr, Ti, and acmite are negligible. Zoisite forms thin blades with tens of *μ*m in length that are close to pure end‐member composition, with <3 mol.% pistacite content. Zoisite in more recrystallized samples is slightly more Fe rich (Tropper & Essene, 1999). Coexisting potassic feldspar is texturally similar to jadeite and lies on the albite–K‐feldspar binary, close to the KAlSi_3_O_8_ end‐member with a mole fraction of 0.91–0.97 (Tropper & Essene, 1999).

#### 3.1.2 Reaction between biotite and former plagioclase

At the interface of biotite and former plagioclase (Figure 1a), a complementary corona involving biotite | garnet ± quartz | phengite–quartz | plagioclase (now pseudomorphs) is developed (b–b*′* in Figure 1b). Biotite forms subhedral mm‐sized flakes and is locally replaced by thin blades and patches of phengite (ph I; Figure 1a–d). Biotite is chemically homogeneous in all textural settings, but is interpreted as compositionally reset during metamorphism (Rubbo et al., 1999). The authors report $X_{\text{Fe}}$ $\left(=\frac{Fe^{2+}}{Fe^{2+}+Mg}\right)$ of 0.45 and Ti contents of 0.29–0.36 a.p.f.u. Phengite (ph I) replacing biotite has elevated TiO_2_ (up to 3 wt%) and high Si contents (3.25–3.40 a.p.f.u.; Rubbo et al., 1999; Tropper & Essene, 1999). The garnet coronae have a homogeneous thickness of ∼50–60 *μ*m and may host patches of vermicular quartz. Garnet forms euhedral crystal faces towards the bimineralic phengite–quartz symplectite and/or matrix quartz (Rubbo et al., 1999). Garnet chemistry is dominated by almandine with low spessartine, but the coronae are asymmetrically zoned with respect to Ca and Fe/Mg. Starting from the biotite side, a typical corona of ∼60 *μ*m thickness shows relatively homogeneous mole fractions of almandine (alm; 0.60–0.70), pyrope (py; 0.15–0.20), grossular (grs; 0.05–0.15), and spessartine (sps; 0.02–0.03) over ∼50 *μ*m of the total thickness. However, within ∼10 *μ*m towards the former plagioclase, the garnet chemistry changes dramatically with an exponential decrease of almandine and pyrope to about 0.45 and 0.05, respectively, whereas grossular increases to 0.45 (figure 2a in Bruno & Rubbo, 2006). Spessartine remains unvaried. Chemical mapping by Rubbo et al. (1999) and electron‐microprobe analyses by Tropper and Essene (1999) show a similar trend. The bimineralic phengite (ph II)–quartz coronae have a comparable thickness, with individual anhedral grains of tens‐ to hundreds *μ*m in size. Phengite in symplectites (ph II) is poor in Ti (<0.1 a.p.f.u.) but is otherwise comparable with phengite replacing biotite (ph I; Rubbo et al., 1999). Minute grains of rutile are occasionally found within the textures.

#### 3.1.3 Reaction between biotite and K‐feldspar

Between biotite and igneous K‐feldspar, a similar composite corona is formed, with the textural architecture of biotite | garnet ± quartz | phengite–quartz | K‐feldspar (c–c*′* in Figure 1c; Biino & Compagnoni, 1992; Bruno et al., 2001). The garnet coronae have comparable thickness and texture but differ in terms of their compositional zoning. Garnets at K‐feldspar sites are significantly less calcic compared to those in contact with former plagioclase and show less pronounced compositional variations (Rubbo et al., 1999). Almandine and pyrope mole fractions plateau at ∼0.70–0.80 and 0.20, respectively. Grossular shows a marked variation towards K‐feldspar, increasing from <0.05 to about 0.15 over the last ∼20% of the profile (Rubbo et al., 1999). Biotite is variably replaced by phengite (ph I), which is compositionally similar to phengite in symplectites (ph II) and the one related to plagioclase replacement textures. Igneous K‐feldspar, up to several cm in size, is close to the orthoclase end‐member composition (Oberhänsli et al., 1985).

#### 3.1.4 Reaction between biotite and quartz

The contact between magmatic biotite and quartz is occupied by a homogeneous monomineralic moat of garnet (∼50 *μ*m; d–d*′* in Figure 1d). Crystals show subhedral faces towards quartz and are more irregular on the biotite side. Compositional data were not reported for this type of corona, but it is expected to be only weakly zoned as documented for nearly identical textures from the Dora Maira sample (see below).

### 3.2 Dora Maira

#### 3.2.1 Former plagioclase site

The original plagioclase is completely replaced by a fine‐grained intergrowth of jadeite–zoisite–K‐feldspar–quartz–kyanite (Figure 1e,f; Biino & Compagnoni, 1992; Bruno et al., 2001). Sodic pyroxene forms small (5–20 *μ*m) prisms and is on average rich in jadeite mole fraction (0.86) with minor Ca‐Eskola (0.13) and Ca‐Tschermak (0.01 Bruno et al., 2001; 2002). Clinopyroxene is generally closer to the jadeite end‐member at the cores of pseudomorphs, whereas it is slightly enriched in Ca at the rims where the modal amount of zoisite is reduced (Biino & Compagnoni, 1992). Zoisite and kyanite form variably‐sized needles (<50 *μ*m) and are close to pure end‐member composition. K‐feldspar forming anhedral grains and patches is dominated by orthoclase (or; ∼90 %) and minor albite (ab) component. Ca is not detected (Bruno et al., 2001). Quartz forms interstitial blebs.

#### 3.2.2 Reaction between biotite and former plagioclase

Biotite in this setting is commonly replaced by phengite (ph I, Figure 1e,f) and may be decorated by minor rutile (Biino & Compagnoni, 1992; Bruno et al., 2001). Former biotite grains are surrounded by a homogeneous corona of garnet (∼40–60 *μ*m) that commonly forms patchy symplectic intergrowths with quartz (Figure 1e,f). Towards former plagioclase, it is followed by a bimineralic corona of phengite (ph II)–quartz (Figure 1e; Biino & Compagnoni, 1992) or intergrowths of garnet–jadeite (Figure 1f; Bruno et al., 2001). In the latter case, symplectites of phengite–quartz may be found in the texture (Figure 1f). The typical mineral association observed is (former) biotite | garnet ± quartz | phengite–quartz ± jadeite | plagioclase (pseudomorphed; see profiles in Figure 1e,f). Small garnet grains commonly overgrow the symplectites (g II in Figure 1e,f; Biino & Compagnoni, 1992; Bruno et al., 2001). Phengite after biotite (ph I) is rich in Ti (0.07–0.19 a.p.f.u.) and shows elevated Si contents between 3.30–3.49 a.p.f.u. (Biino & Compagnoni, 1992). Garnet, forming subhedral faces away from the phengite/former biotite, shows strong compositional gradients from the biotite side (alm_73_py_16_grs_3_sps_3_) towards the plagioclase side (alm_45_py_6_grs_43_sps_1_), whereas garnet overgrowing symplectites (g II) is rich in grossular (60–70 %; Biino & Compagnoni, 1992). Jadeite towards former plagioclase is compositionally similar to pyroxene within the pseudomorphs (Bruno et al., 2001).

#### 3.2.3 Reaction between biotite and K‐feldspar

At the interface between igneous biotite and K‐feldspar a complementary corona of garnet ± quartz (10–120 *μ*m) at the biotite side and symplectic phengite (ph II)–quartz (∼100 *μ*m) at the K‐feldspar side is developed (Figure 1g; Bruno et al., 2001). Biotite is replaced by phengite (ph I) to variable extent. The garnet coronae have roughly homogeneous compositions, with alm_78 − 80_py_15 − 17_grs_3 − 4_sps_1 − 2_ (Biino & Compagnoni, 1992; Bruno et al., 2001). The adjacent phengite (ph II) intergrown with quartz is free of Ti and rich in Si (3.28–3.40 a.p.f.u.; Biino & Compagnoni, 1992). Precursor K‐feldspar forms subhedral prisms (max. ∼2–3 cm) and shows perthitic exsolutions and inclusions of the other igneous minerals. It is assumed to be compositionally reset, with or_90_ab_10_ (Bruno et al., 2001). Recalculated igneous compositions range between or_70 − 75_ab_25 − 30_ (Biino & Compagnoni, 1992).

#### 3.2.4 Reaction between biotite and quartz

Igneous biotite is separated from quartz by a homogeneous garnet moat with a thickness between 5 to 40 *μ*m (d–d*′* in Figure 1g). The original biotite is either compositionally reset or replaced by phengite (ph I) to variable degrees (Figure 1g; Biino & Compagnoni, 1992; Bruno et al., 2001). Garnet in the moats is compositionally roughly homogeneous, with alm_76 − 78_py_21 − 23_grs_1 − 2_sps_1 − 2_. Igneous quartz is now recrystallized showing a polygonal granoblastic texture, interpreted as former coesite by Biino and Compagnoni (1992).

### 3.3 Iberian Massif (MTU)

Ibarguchi (1995) describes a suite of rocks with coronitic textures, ranging from granitic to granodioritic composition. The textures in the various samples are similar and schematically summarized in Figure 1h.

#### 3.3.1 Former plagioclase site

Former plagioclase (∼0.5–1 cm in diameter) is completely replaced by pseudomorphs of fine‐grained albite–zoisite–phengite ± jadeite. Minute needles of kyanite may be present, and quartz is rarely reported in jadeite–albite‐bearing textures. Clinopyroxene is rich in jadeite (∼86–92 mol.%) but becomes slightly enriched in FeO (max. ∼5.50 wt%) in the vicinity of rare inclusions of igneous magnetite–ulvospinel within the former plagioclase (Ibarguchi, 1995). Albite and zoisite approach end‐member compositions, with ab_86 − 98_ and ∼1 mol.% pistacite content, respectively. Phengite has ∼3.25 Si a.p.f.u. and negligible Ti.

#### 3.3.2 Reaction between biotite and former plagioclase

Igneous biotite appears partly replaced by blades, patches, or rims of phengite ± rutile (Ibarguchi, 1995). Biotite is surrounded by a homogeneous moat of garnet up to ∼100 *μ*m thick, with crystals forming euhedral faces away from biotite. It is followed by symplectites involving phengite ± quartz and occasionally albite towards the former plagioclase side. Relic biotite has $X_{\text{Fe}}$ of ∼0.4–0.5 and is rich in TiO_2_ (3–5 wt.%), whereas the phengite overgrowing it is enriched in the latter (1–2 wt.%) with up to 3.40 Si a.p.f.u. (Ibarguchi, 1995). Garnet in the coronae shows asymmetric chemical zoning towards former plagioclase, with a strong increase in grossular mole fraction from about 10 to 40 and concomitant decrease in almandine and pyrope (∼75 to 55 and 15 to 5, respectively; Ibarguchi, 1995).

#### 3.3.3 Other reaction textures

Reaction texture between igneous biotite and K‐feldspar or quartz are not described in detail by Ibarguchi (1995); however, the author points out that igneous biotite is always rimmed by moats of garnet which may be followed by composite symplectic coronae of phengite, albite, and/or quartz.

### 3.4 Summary

In all cases, the most prominent texture involves the complete pseudomorphic replacement of igneous plagioclase by jadeite–zoisite–K‐feldspar–quartz ± kyanite (Western Alps) and albite–jadeite–zoisite–phengite ± kyanite ± quartz (Iberian Massif). Igneous biotite may be replaced by phengite to variable extent. Between biotite and former plagioclase, a double‐layered corona involving (original) biotite | garnet ± quartz | phengite–quartz ± jadeite | plagioclase (pseudomorphed) is found. Biotite and K‐feldspar typically show a reaction interface with biotite | garnet ± quartz | phengite–quartz | K‐feldspar. Garnet at biotite–feldspar boundaries shows subhedral crystal faces away from biotite, suggesting it grew at the expense of phengite–quartz symplectites. Finally, between igneous biotite and quartz, the typical arrangement is biotite | garnet | quartz.

## 4 PETROLOGICAL INTERPRETATION

All observed textures are characterized by a high degree of spatial organization and are confined to specific locations, in particular interfaces separating former igneous minerals. During high‐pressure overprint of the granitic equilibrium assemblages, limited chemical communication caused the coarse‐grained igneous minerals to develop into distinct compositional domains, which in turn induced the growth of specific metamorphic mineral assemblages at their interfaces (‘mosaic’ equilibrium; Khorzhinskii, 1959). The textures are therefore interpreted to reflect local equilibria, with limited exchange having occurred across the different compositional domains. Corona textures and pseudomorphs are commonly regarded as retrograde features that form when high‐grade rocks slowly cool and/or decompress from peak metamorphic conditions. This is particularly the case in granulites (Doukkari et al., 2018; Schorn et al., 2020; White & Powell, 2011) and eclogites (Baldwin et al., 2015; Štípská et al., 2014; Vrabec et al., 2012). Cooling of fluid‐ and/or melt‐absent, coarse‐grained assemblages hampers lower grade equilibration and favours the build‐up of chemical potential ‘landscapes’, which are flattened in time via diffusion in an attempt to restore equilibrium at the new *P*–*T* conditions. Because chemical components have contrasting diffusivities, more or less complex corona and pseudomorphic textures develop, separating two or more metamorphic minerals that are in disequilibrium at the new *P*–*T* conditions (Powell et al., 2019; White et al., 2008).

Based on their relative diffusivities, elements are categorized into three groups: (i) essentially immobile elements with variable chemical potentials across the reaction texture, (ii) highly mobile elements whose chemical potentials can be assumed as constant and superimposed across the compositional domain, and (iii) elements that are mobile across the texture but whose chemical potentials are variable at this scale. Al is commonly considered to be effectively immobile at the sub‐cm‐scale, at least in the absence of fluid. Similarly, strongly‐bonded cations such as Si and the rare earth elements remain immobile up to the lower granulite facies (Carlson, 2002). Small cations, in particular H_2_(O), have high diffusivities and fall into the second group of elements. These are typically sourced from outside the site of texture formation and are considered to not have influenced its evolution. This assumption may be justified for granulite facies textures (e.g., Doukkari et al., 2018; Schorn et al., 2020); however, this is not the case for reactions that are explicitly caused by the addition of H_2_(O), such as those related to the gabbro‐to‐eclogite transition (Schorn & Diener, 2017). Finally, Fe, Mg, and less so Ca are part of group (iii), whose gradients are considered as the textural driving forces and used for their graphical representation (as axes) in chemical potential diagrams (e.g., Doukkari et al., 2018; White et al., 2008).

The texture‐forming processes described here are somewhat different to the retrograde scenario because they are considered a prograde feature related to the incipient attempt of the granitoids to equilibrate at eclogite facies conditions (Bruno et al., 2001; Rubbo et al., 1999). The mechanisms by which incipient eclogitization occurred are considered similar to those described by Schorn and Diener (2017) for the eclogite‐type locality (Austria). There, dry gabbros persisted metastably at high‐pressure conditions until infiltration of exogenous fluid triggered the partial eclogitization of the rocks (cf. Austrheim, 1987; Wayte et al., 1989; Wain et al., 2001). Similar to the metagranitoids described here, the main textural developments in the metagabbros of Schorn and Diener (2017) involve the complete breakdown of igneous plagioclase and the formation of various coronae. Because there it occurred below the albite = jadeite + quartz transition, the original plagioclase is pseudomorphed by a fine‐grained mesh of albite–(clino‐)zoisite–kyanite ± quartz. The textures developed in response to the (im)mobility of different components on variable length scales, with the plagioclase pseudomorphs forming via transfer of H_2_O into the texture site. The incoming H_2_O was accommodated in hydrous (clino‐)zoisite forming along with anhydrous products at the expense of plagioclase, but all other elements remained effectively immobile over the scale of this texture (Schorn & Diener, 2017). The H_2_O required for driving eclogitization of the gabbros was derived from nearby metapelites (Schorn, 2018), whereas the metagranitoids described here are considered a closed system, with H_2_O solely hosted in igneous biotite of the protoliths (e.g., Ibarguchi, 1995). I consider that partial breakdown of biotite occurred at some point during prograde burial and heating, with the liberated H_2_O diffusing outwards and into nearby anhydrous minerals down gradients in chemical potential (*μ*H_2_O). The enhancing effect of H_2_O on reaction rates—even if restricted to tens of ppm H_2_O (Milke et al., 2013)—and simultaneous transfer of other components (Fe–Mg and Na–Ca) among compositional domains caused texture formation at the sites affected by H_2_O transfer. I will show here that it was indeed the relative mobility of H_2_O compared to other elements that controlled the textural architecture in the various samples and that the particular *P*–*T* evolution at each study location played a subordinate role.

### 4.1 Different textures at different scales

The main mineral reaction textures in the metagranitoids from the study sites in this paper occur over two contrasting length scales, similar to the microstructures in eclogitized gabbros (Schorn & Diener, 2017). This observation suggests that the formation of the textures was controlled by elements of contrasting diffusive mobility. The former plagioclase sites from the Iberian Massif rocks are up to ∼1 cm in size (Ibarguchi, 1995), and similar to the other investigated samples, they are pervasively replaced by metamorphic minerals (Biino & Compagnoni, 1992; Bruno et al., 2001; Bruno & Rubbo, 2006; Compagnoni & Maffeo, 1973; Ibarguchi, 1995; Oberhänsli et al., 1985; Rubbo et al., 1999; Tropper & Essene, 1999). This indicates that the main diffusing component(s) responsible for their formation were mobile (at least) over this scale. In contrast, all observed coronae textures are only a few hundreds of *μ*m in thickness, implying that their controlling elements were mobile on a significantly smaller scale. Unlike the plagioclase pseudomorphs that developed in relative chemical isolation, the coronae (e.g., between biotite–plagioclase or biotite–K‐feldspar) are complementary, suggesting a more complex, coupled diffusion of elements among distinct compositional domains. The textures are therefore modelled separately, as outlined in Schorn and Diener (2017).

## 5 PHASE EQUILIBRIUM MODELLING

Phase diagrams were calculated using thermocalc v3.45 (Powell & Holland, 1988) and an updated version of the Holland and Powell (2011) data set (file tc‐ds62.txt, created 06/02/2012). The used activity–composition models are diopside–omphacite–jadeite of Green et al. (2016); muscovite–paragonite of White, Powell, Holland, et al. (2014); biotite, garnet, and orthopyroxene of White, Powell, and Johnson (2014); and plagioclase–K‐feldspar of Holland and Powell (2003). The aluminosilicates, albite, quartz, zoisite, and H_2_O are pure end‐member phases. Phase abbreviations are those used by thermocalc and are as follows: albite (ab), aluminosilicate (als), andalusite (and), aqueous fluid (H_2_O), biotite (bi), clinopyroxene (cpx), diopside (dio), garnet (g), grossular (gr), jadeite (jd), K‐feldspar (ksp), kyanite (ky), muscovite (mu), omphacite (o), phengite (ph), plagioclase (pl), quartz (q), sillimanite (sill), and zoisite (zo). Phase proportion plots (‘modeboxes’) were constructed using unpublished Mathematica scripts (R. Powell, personal communication), where phase abundances were calculated as mole fractions, with each phase normalized to one oxide sum total to approximate volume percent.

### 5.1 A simple petrogenetic grid

**FIGURE 2:**
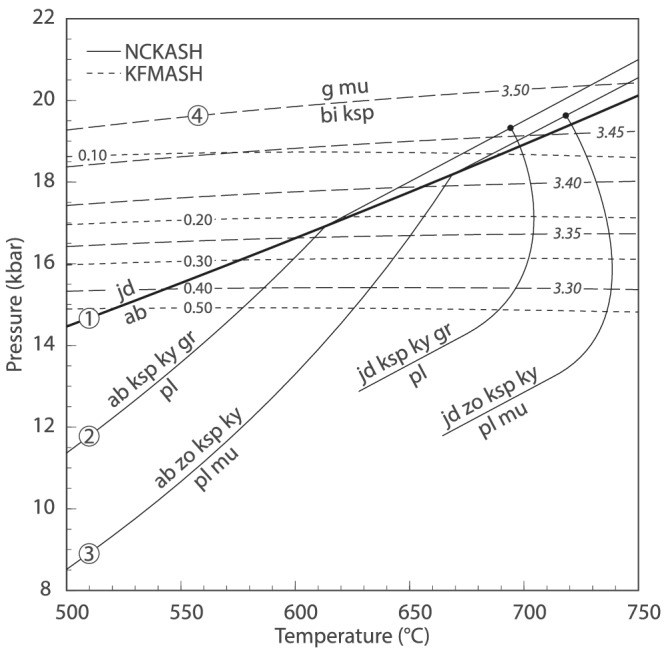
Calculated P– T projection showing key reactions for plagioclase‐related textures in the NCKASH model system and biotite–K‐feldspar interfaces in KFMASH. The latter are calculated using a fixed $X_{\text{Fe}}$ of 0.40 in biotite and variable Si‐in‐phengite (long dashes, italic labels) and at various biotite‐ $X_{\text{Fe}}$ but fixed Si‐in‐phengite of 3.30 a.p.f.u. (short dashes). Quartz is in excess

A simple *P*–*T* projection, calculated in two separate model systems, is presented in Figure 2. The pseudomorphs after plagioclase involve jadeitic clinopyroxene, zoisite, K‐feldspar, kyanite, and quartz in the Western Alps rocks, and albite, jadeite, zoisite, phengite, and kyanite ± quartz in the Iberian samples. Because sodic pyroxene, zoisite, albite, and kyanite are close to their end‐member compositions, they can be represented as pure phases in the simplified Na_2_O–CaO–K_2_O–Al_2_O_3_–SiO_2_–H_2_O (NCKASH) model system. Within this composition, garnet and phengite are end‐member grossular and muscovite, respectively. Only the feldspars allow for Na–Ca–K solid solution.

The degenerate univariant reaction

(1) albite=jadeite+quartz

defines a *P*–*T* space where albite is stable or jadeite coexists with quartz. As such, the reactions

(2) $$\begin{array}{cc} & \mathrm{plagioclase}\;=\;\mathrm{albite}/\mathrm{jadeite}+\text{K‐feldspar}+\text{kyanite}\\ & \;+\;\mathrm{grossular}\;+\;\mathrm{quartz} \end{array}$$

and

(3) $$\begin{array}{cc} & \mathrm{plagioclase}\;+\;\mathrm{muscovite}\;=\;\mathrm{albite}/\mathrm{jadeite}+\mathrm{zoisite}\;+\\ & \text{K‐feldspar}+\mathrm{kyanite}\;+\;\mathrm{quartz} \end{array}$$

involve albite or jadeite at low‐ and high‐pressure conditions, respectively (Figure 2). Note that reaction (2) is degenerate. The crossing of reactions (2) and (3) towards high pressure is responsible for the observed plagioclase pseudomorphs, as explained in detailed below.

The complementary symplectites separating biotite from K‐feldspar and quartz additionally involve garnet and phengite. Because other components are minor, these textures can be simulated in the K_2_O–FeO–MgO–Al_2_O_3_–SiO_2_–H_2_O (KFMASH) model system. Garnet, muscovite, and biotite have solid solutions in KFMASH, whereas K‐feldspar is end‐member orthoclase. In order to graphically represent the reaction involving all reactants and products, a constant $X_{\text{Fe}}$ in biotite of 0.40 was assumed, based on the compositions reported in the textures. The trivariant reaction

(4) garnet+muscovite+quartz=biotite+K‐feldspar

is calculated at different Si‐in‐phengite contents to showcase the effect on the *P*–*T* stability of the symplectite assemblages (long‐dashed lines on Figure 2). In order to monitor variable biotite composition, the same reaction is calculated at a fixed Si‐in‐phengite of 3.30 a.p.f.u. but different biotite‐*X*_Fe_ (short‐dashed lines on Figure 2). Reaction (4) is relatively temperature‐insensitive at fixed $X_{\text{Fe}}$ in biotite and Si‐in‐phengite but is shifted towards higher pressure with decreasing biotite‐*X*_Fe_. An increase of Si‐in‐phengite has the same effect. It is shown below that the choice of biotite and phengite chemistry has a subordinate impact on phase relations, albeit affecting the absolute location of the reaction in *P*–*T* space. It is pointed out that reaction (4) is a simplified version of the multivariant reaction(s) involved in the actual biotite breakdown. As such, it is used here to qualitatively reproduce the potential phase relations involved in the formation of the textures. In general, shifting conditions towards higher pressure causes (igneous) biotite and K‐feldspar to become unstable in favour of garnet and phengite‐rich muscovite, as found in the coronae.

### 5.2 Reaction textures replacing plagioclase

The pseudomorphs described here (e.g., Figure 1a,e) are similar to textures replacing igneous plagioclase in eclogitized gabbros (Schorn & Diener, 2017; Wayte et al., 1989), where plagioclase breakdown was essentially isochemical with the exception of H_2_O being added from outside the site of texture formation. Unlike in gabbros, plagioclase in granitoids contains significant potassium, which is considered in the modelling here.

**FIGURE 3:**
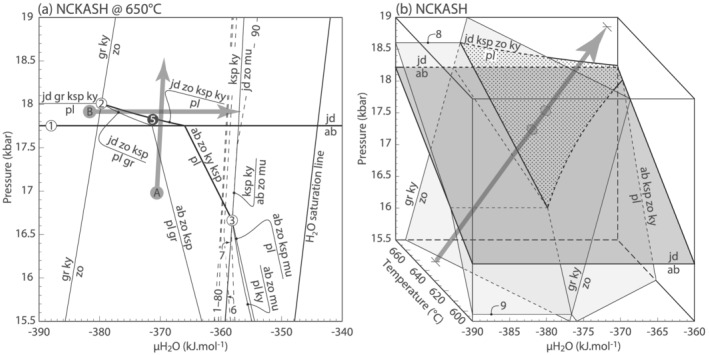
NCKASH phase relations for plagioclase pseudomorphs. (a) Isothermal P– μH_2_O diagram calculated at 650°C. Dashed lines are contours for H_2_O content as percentage of the amount required for saturation. Thick‐numbered reactions and potential trajectories related to textural development (arrows) are discussed in the text. Reaction 6 is [zo]: ksp + ky = pl + mu + ab; Reaction 7 is [ab]: ksp + zo + ky = pl + mu with right‐hand side referring to high μH_2_O. (b) Partial P– T– μH_2_O diagram showing main reactions of (a). Point (3) and the [ky] reaction of point (2) are omitted for simplicity. Dotted area refers to reaction (5), crossed with increasing pressure and μH_2_O and/or with decreasing temperature (arrow). Reactions 8 and 9 are [zo] of point (2) as labelled on (a), but with jadeite and albite at high and low pressure, respectively. Quartz is in excess

Figure 3a is an isothermal *P*–*μ*H_2_O diagram computed in the NCKASH compositional system at 650°C. As such, degenerate reaction (1) becomes an isobaric line whereas reactions (2) and (3) correspond to effective invariant points (numbers in white dots). Because *μ*H_2_O is explicitly considered as axis, reaction (2) is no longer degenerate and the point now also involves zoisite. Shown are only [H_2_O] reactions because H_2_O‐present ones are collinear with the H_2_O saturation line. Analogously, reactions involving albite–jadeite–quartz overlap with reaction (1). Because reaction (2) involves only one hydrous mineral, zoisite, it is intersected at lower *μ*H_2_O compared to reaction (3) which additionally involves muscovite. At the chosen temperature, reaction (2) involves jadeite whereas reaction (3) hosts albite instead. Similarly, lines that cross reaction (1) involve albite or jadeite at low and high pressure, respectively. Plagioclase is stable at the low‐pressure and ‐*μ*H_2_O side of reaction lines.

The plagioclase breakdown textures found in the metagranitoids formed according to the reaction

(5) $$\begin{array}{cc} & \mathrm{plagioclase}\;+\;H_{2}O\;=\;\mathrm{jadeite}\;+\;\mathrm{zoisite}\;+\;\mathrm{kyanite}\;+\;\\ & \text{K‐feldspar}+\text{quartz} \end{array}$$

which corresponds to the NCKASH variant of the reaction proposed by Wayte et al. (1989), with additional K‐feldspar as product. Reaction (5) is the respective [gr]‐ and [mu] line connecting the points of reactions (2) and (3), labelled with a white number in a black dot on Figure 3a. The same reaction, but involving albite instead of jadeite in the K_2_O‐free system, is responsible for plagioclase replacement in metagabbros of the eclogite‐type locality (Schorn & Diener, 2017).

Contours for percentage of H_2_O saturation overlain on Figure 3a demonstrate that the textures formed at very low H_2_O contents (<1% of saturation), and a free fluid was not involved in texture formation. Closed‐system pressure increase leads to a concomitant increment in *μ*H_2_O (trajectory A on Figure 3a), causing the crossing of reaction (5) without necessarily requiring additional increase in *μ*H_2_O via, for example, influx of H_2_O (trajectory B). Pressure increment causes the consumption of precursor plagioclase to produce the jd–zo–ksp–ky–q intergrowths, whereas increase of *μ*H_2_O leads to the consumption of K‐feldspar and kyanite from the textures to produce additional jadeite, zoisite, and muscovite (trajectory B; Figure 3a) as observed in samples from Monte Mucrone (Tropper & Essene, 1999). Some textures may involve both jadeite and albite together with phengite (e.g., Ibarguchi, 1995), although the petrographic descriptions therein are not clear if jadeite and albite coexisted at equilibrium, nor if quartz was part of the assemblage. Assuming the former applied, quartz should not be part of the equilibrium, and the textures have formed via crossing of a [q]‐variant of reaction (3). Alternatively, as suggested by Ibarguchi (1995), jadeite–quartz stability was reached during the prograde, and albite ± muscovite are retrograde products that may have formed during cooling and/or decompression at the expense of sodic clinopyroxene (e.g., Biino & Compagnoni, 1992; Bruno et al., 2001; Compagnoni & Maffeo, 1973; Oberhänsli et al., 1985).

It should also be noted that choice of temperature at which Figure 3a was calculated is somewhat arbitrary. Importantly, however, regardless of the exact *P*–*T* conditions, the reaction topology remains unvaried except for the participation of albite instead of jadeite, as shown in Figure 3b. Because the investigated textures mostly involve the latter, they must have developed at *P*–*T* pairs above reaction (1), for example, along the *P*–*T*–*μ*H_2_O path drawn in Figure 3b. Crossing of reaction (5) may be achieved via compression, an increase of *μ*H_2_O and/or cooling, but it is unlikely that the latter played a significant role in light of the prograde nature of the textures (Bruno et al., 2001; Bruno & Rubbo, 2006).

### 5.3 Coronae between biotite–quartz and biotite–K‐feldspar

Biotite is separated from quartz by a simple layer of garnet (Figure 1d,g), whereas between K‐feldspar, the complementary corona biotite | garnet ± quartz | phengite–quartz | K‐feldspar is found (e.g., Figure 1c). The formation of garnet indicates the transport of FeO–MgO and possibly the removal of H_2_O from biotite. Similarly, the phengite–quartz symplectites growing at the expense of K‐feldspar require the intake of H_2_O, FeO, and MgO.

**FIGURE 4:**
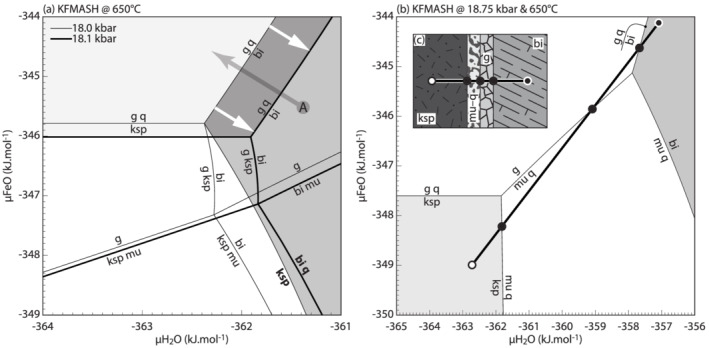
KFMASH chemical potential diagrams for biotite–K‐feldspar textures calculated at fixed P– T on the (a) right‐hand side and (b) left‐hand side of reaction (4). White arrows highlight shift in phase relations with pressure. Grey arrow indicates a chemical potential trajectory discussed in the text. Diagrams are calculated at fixed μK_2_O and μMgO of −842.77 and −661.83 kJ.mol^−1^, respectively. Only quartz‐present equilibria are shown in (b). (c) Comic showing the texture at biotite–K‐feldspar interfaces

Chemical potential diagrams for KFMASH reaction (4) calculated at 650°C and within the stability field of jadeite + quartz (Figure 2), are presented in Figure 4. In order to preserve the variance of the reaction, *μ*K_2_O and, in a first instance, *μ*MgO were fixed at values calculated for reaction (4) at 650°C and ∼18.35 kbar. Because the stability of reaction (4) is dependent on mineral composition (Figure 2), several combinations of *P*–*T*–*μ*K_2_O–*μ*MgO may be chosen without altering the phase diagram topology. Fixing chemical potentials implies that their values are constant and superimposed over the scale of the texture, effectively rendering them perfectly mobile. Typically, H_2_(O) is the preferred candidate due to its high diffusivity, but in this case, *μ*H_2_O plays a crucial role in controlling textural development and cannot be considered as constant over the texture. Due to the potassic phases involved (biotite, phengite, and K‐feldspar), *μ*K_2_O is considered as unlikely to have affected the first‐order phase relations and was chosen as superimposed chemical potential. Fixing *μ*MgO is a similar simplification but is required in order to graphically represent the phase relations on a two‐dimensional diagram (Figure 4). Setting *μ*FeO instead has the equivalent effect and does not alter the diagram topology. As such, variations in *μ*FeO–*μ*MgO can be regarded as coupled, as is the case in the textures. Al_2_O_3_ and SiO_2_ are taken as immobile, and *μ*H_2_O and *μ*FeO explicitly quantified as axes.

Figure 4a shows the phase relations on the right‐hand side of reaction (4), calculated at 650°C and two different pressures to simulate isothermal burial prior to crossing the reaction. K‐feldspar and muscovite are stable at low *μ*H_2_O–*μ*FeO, with garnet present at high *μ*FeO (light grey shading) and biotite calculated at high *μ*H_2_O (dark grey shading). The stable igneous assemblage of K‐feldspar–biotite–quartz is calculated along the [mu] line emanating towards low *μ*FeO/high *μ*H_2_O (bold labels; Figure 4a). Increasing the pressure (from 18 to 18.1 kbar) preserves the diagram topology but causes an overall shift of phase relations towards higher *μ*H_2_O and less so, lower *μ*FeO (white arrows on Figure 4a). This causes an expansion of the garnet stability field, and former biotite domains now lie within garnet–quartz stability (overlapping area on Figure 4a). The monomineralic garnet coronae can therefore form directly on biotite due to changing *P*(–*T*) conditions during burial of the rocks. Alternatively, at fixed *P*–*T*, the bi–g–q line can be crossed via diffusion of H_2_O away from biotite, towards lower *μ*H_2_O and higher *μ*FeO (path A; Figure 4a).

Once reaction (4) is crossed towards higher *P*, a topological inversion occurs (Figure 4b). K‐feldspar is confined to low *μ*H_2_O–*μ*FeO whereas biotite is stable at high *μ*H_2_O. The intermediate compositional space is occupied by muscovite–quartz and garnet ± quartz at low and high *μ*FeO, respectively (Figure 4b). The textural architecture (e.g., Figure 1c and schematically drawn in Figure 4c) is reproduced along the suggested *μ*H_2_O–*μ*FeO trajectory, corresponding to H_2_O–FeO transfer from biotite into K‐feldspar sites down gradients of the respective chemical potential.

**FIGURE 5:**
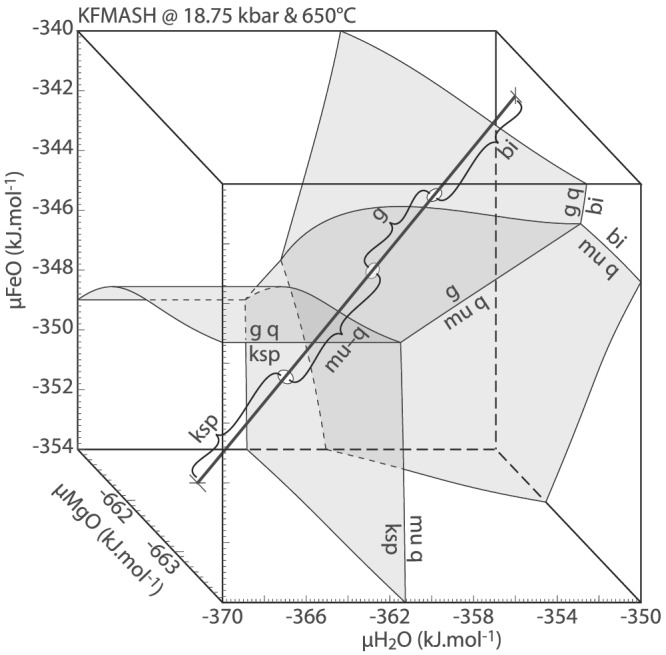
μH_2_O– μMgO– μFeO diagram for Figure 4b, calculated at μK_2_O = −842.77 kJ.mol^−1^. Thick black line is a possible chemical potential vector (see text). Only quartz‐bearing equilibria are shown

In order to show the effect of coupled H_2_O–FeO–MgO diffusion, a three‐dimensional box diagram involving *μ*H_2_O–*μ*FeO–*μ*MgO for the left‐hand side of reaction (4) is drawn (Figure 5). Figure 4b discussed earlier corresponds to a section at constant *μ*MgO through this compositional volume. However, reaction lines now correspond to areas and phase stability fields are volumes in Figure 5. The textural arrangement biotite | garnet | muscovite–quartz | K‐feldspar follows a composite *μ*H_2_O–*μ*FeO–*μ*MgO vector (Figure 5). The interfaces that separate mineral coronae correspond to the compositional trajectory piercing through reaction surfaces (compare to Figure 4b,c), suggesting that coupled diffusion of all three components—with possible minor contribution of K_2_O—from biotite to K‐feldspar is required to form the textures.

### 5.4 Coronae separating biotite and former plagioclase

**FIGURE 6:**
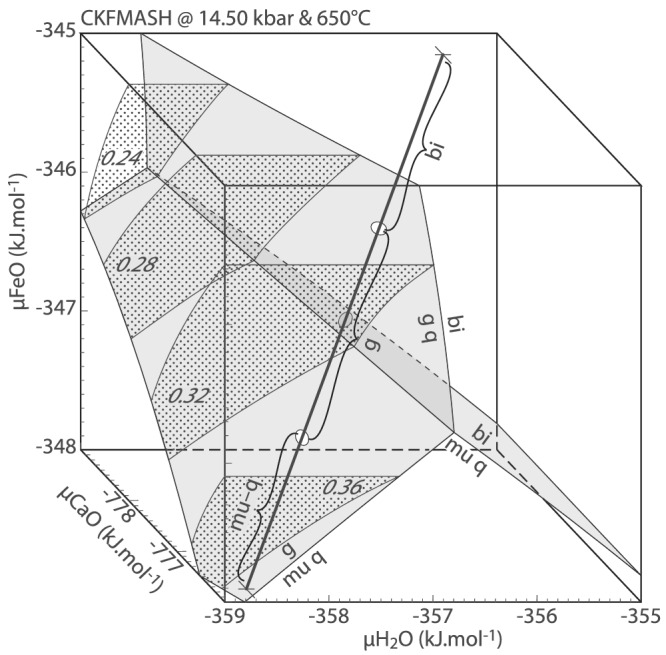
Partial μH_2_O– μCaO– μFeO diagram for biotite–plagioclase textures calculated with fixed μK_2_O of −859.54 and μMgO of −670 kJ.mol^−1^ in CKFMASH. Dotted areas are contours for grossular mole fraction in garnet. Thick black line is a chemical potential vector discussed in the text. Quartz is in excess

The textures in this setting are essentially analogous to the ones described above, with a composite corona of symplectic phengite–quartz and garnet developed between plagioclase pseudomorphs and biotite (e.g., Figure 1a,b,e). Patches and/or layers of jadeite may be found on the plagioclase side (Figure 1f) and are considered as part of the plagioclase pseudomorphs. The overall similarity to biotite–K‐feldspar‐related textures suggests that they are controlled by similar phase relations and diffusion of the same main components. Unlike in textures next to potassic feldspar, the garnets in this setting show significant compositional zoning, in particular with a strong increase of Ca towards former plagioclase (e.g., Bruno & Rubbo, 2006). The model system is thus expanded to CaO–K_2_O–FeO–MgO–Al_2_O_3_–SiO_2_–H_2_O (CKFMASH), and the phase relations responsible for texture formation are shown in Figure 6. Garnet is now a ternary almandine–pyrope–grossular solid solution, and muscovite additionally contains a margarite component.

The diagram is calculated for the K‐feldspar‐absent point of reaction (4), shifted towards lower pressure due to the additional component. In order to draw a three‐dimensional box diagram, both *μ*K_2_O and *μ*MgO were set at constant values, calculated for the reaction intersected at 650°C. The resulting phase relations are similar to those shown in Figure 5, with biotite stable at high *μ*H_2_O–*μ*FeO and muscovite–quartz calculated at variable *μ*H_2_O and lower *μ*FeO (Figure 6). The consideration of CaO has a limited effect on the phase stability volumes; however, garnet is stabilized towards lower *μ*FeO with increasing *μ*CaO, corresponding to an increase in grossular component (contours on Figure 6). This effect is consistent with the observed Ca zonation of garnet in the vicinity of former plagioclase where *μ*CaO was highest. The proposed *μ*H_2_O–*μ*FeO–*μ*CaO trajectory pierces the compositional volume from the stability field of biotite at high *μ*H_2_O–*μ*FeO to that of muscovite–quartz at lower *μ*H_2_O. The intervening volume is occupied by garnet, showing an increasing grossular content towards muscovite–quartz. Note that plagioclase does not participate in the calculated phase relations because a binary Ca–K‐feldspar is not stable at the conditions of interest. However, it is likely that at the time of texture formation, plagioclase decomposed into the pseudomorphs due to H_2_O flux from biotite (Figure 3), with the effective result of Ca being transported in the opposite direction towards biotite. This would result in the observed asymmetric grossular zoning in garnet (Bruno & Rubbo, 2006).

Biotite also shows variable replacement by phengite, forming patches and/or layers (Figure 1). This type of direct replacement may be explained by a complex vector through compositional space (Figure 6), but it is possible that other components that are not considered in the modelling here (e.g., TiO_2_ and/or Fe_2_O_3_) were responsible. The calculations are carried out in reduced model systems for the sake of simplicity and graphical representation, and as such, they have a limited capability to simulate complex natural processes. Because the first‐order phase relations are adequately reproduced, further investigations of more complex chemical systems are beyond the scope of this article.

## 6 DISCUSSION

### 6.1 Contrasting diffusive length scales of H_2_O?

The pseudomorphs and coronae contain hydrous minerals that formed at the expense of nominally anhydrous precursor plagioclase and K‐feldspar. The textures therefore imply diffusion of H_2_(O). However, the length scales of pseudomorphs (∼cm) and coronae (∼*μ*m) differ over at least two orders of magnitude. H_2_(O) is simultaneously the fastest diffusing component when compared to the other main elements involved in texture formation and is unlikely to selectively diffuse over such contrasting length scales. The diffusivity of H_2_(O) alone therefore cannot explain the different textural scales. Additionally, reaction (5) related to the pseudomorphs apparently went to completion in all instances, with no plagioclase left in the textures (Biino & Compagnoni, 1992; Bruno et al., 2001; Bruno & Rubbo, 2006; Compagnoni & Maffeo, 1973; Ibarguchi, 1995; Oberhänsli et al., 1985; Rubbo et al., 1999; Tropper & Essene, 1999). On the other hand, K‐feldspar is largely preserved and only partially consumed by symplectites. It can therefore not be simply argued that the available H_2_(O) was (in)sufficient to drive the reactions to (in)completion.

As demonstrated above, the observed plagioclase breakdown occurred with only the addition of H_2_O to an otherwise anhydrous system (Figure 3). In contrast to such closed‐system evolution, the coronae involving phengite require the additional coupled diffusion of FeO–MgO(–CaO) into the former feldspar site (Figures 4, 5, 6). Qualitatively, this indicates that in all cases, H_2_(O) diffused the farthest, but the other components required to form the symplectites lagged behind, and phengite could only form once the additional components arrived at the site of reaction. Indeed, the presence of zoisite in plagioclase pseudomorphs suggests that not all H_2_O was consumed by the phengite mantling the pseudomorphs and the incoming H_2_O was sufficient to cause the breakdown of the original plagioclase. The textures therefore provide an example where the coupled diffusion can be documented semi‐quantitatively, with H_2_(O) alone diffusing at the cm‐scale, and coupled H_2_(O)–FeO–MgO(–CaO) transfer being limited to the *μ*m‐scale. In this case, the slowest diffusing component, most likely CaO (Carlson, 2002) or in its absence FeO–MgO, controlled the scale at which the textures developed.

### 6.2 Incipient closed‐system ‘eclogitization’

It is generally accepted that the eclogitization of gabbro or granulite requires the addition of H_2_O (Austrheim, 1987; Gilotti & Elvevold, 1998; Wayte et al., 1989; Wain et al., 2001; Zhang & Liou, 1997), in particular as catalyst for enhancing rates of dissolution–precipitation (e.g., Ahrens & Schubert, 1975; Rubie, 1986). If the protolith is largely anhydrous, H_2_O must be added from outside the system in order to cause reaction, with the precursor surviving metastably in the absence of external fluid infiltration (Ahrens & Schubert, 1975; Schorn & Diener, 2017). Metagranitoids, such as those described here, do not strictly require the addition of exogenous H_2_O because they intrinsically contain hydrous minerals such as biotite. During burial, the partial breakdown of biotite is accompanied by minor release of H_2_O, which in turn causes the breakdown of plagioclase once it diffuses into nearby sites (path B on Figure 3). Even in the absence of additional H_2_O, plagioclase breakdown can occur by mere pressure increase (path A on Figure 3). Note that in all cases, H_2_O contents are extremely low and do not require the participation of a free fluid (Schorn & Diener, 2017). Therefore, provided reactions are crossed, metagranitoids are likely to undergo at least incipient conversion to their eclogite facies counterparts.

**FIGURE 7:**
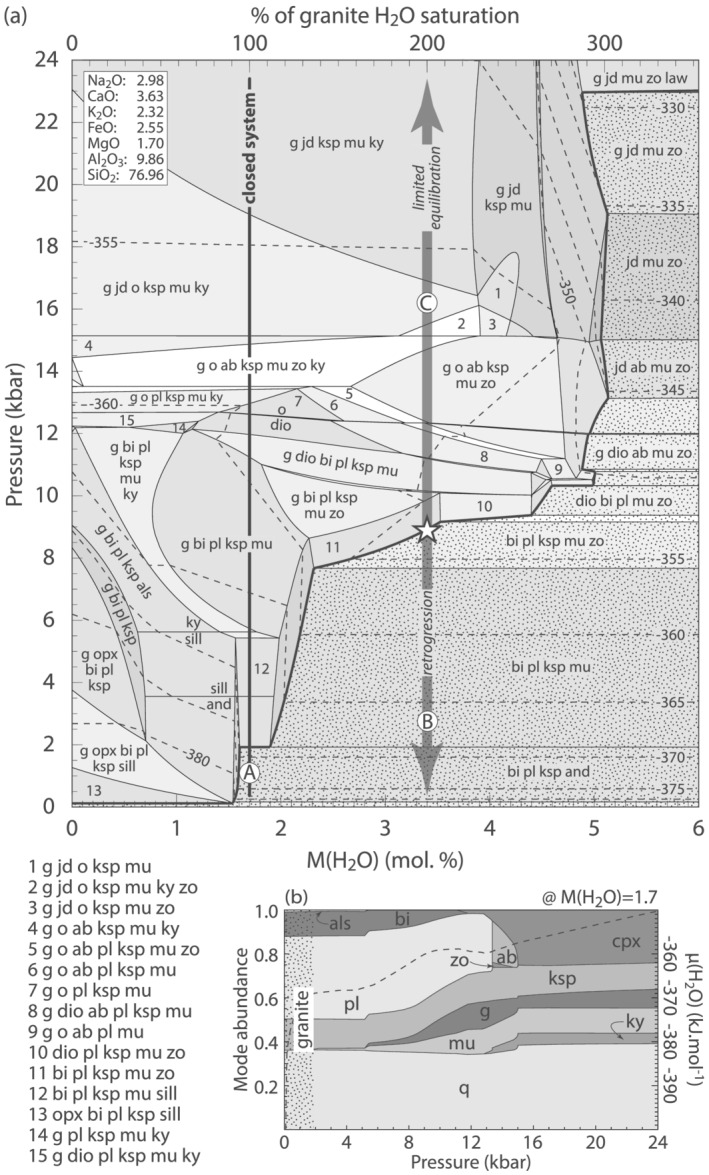
Isothermal phase diagrams calculated for average metagranodiorite in NCKFMASH at 600°C. (a) P– M(H_2_O) pseudosection. The fluid saturation line is in bold with dotted shading indicating H_2_O saturation (H_2_O not labelled). Arrows are discussed in the text. (b) Modebox calculated at low M(H_2_O) = 1.7 mol.% (black line labelled ‘A’ on a). Dashed lines are contours for μH_2_O (kJ.mol^−1^). Bulk composition is in mol.%, quartz is in excess

High‐pressure metamorphism of metagranitoids is simulated with an isothermal *P*–*M*(H_2_O) pseudosection (Figure 7a). Such a diagram implies equilibrium at all times; hence, it is not directly relevant for reaction textures that formed in individual domains of local bulk composition. However, it is useful to showcase general mineralogical trends, in particular the relationship between pressure and *M*(H_2_O) to estimate the conditions of H_2_O saturation, and the potential for equilibration in the presence of fluid. The modelled composition is an average Monte Mucrone metagranodiorite taken from Oberhänsli et al. (1985). The authors presented whole‐rock bulk compositions for a suite of variably transformed samples and concluded that metamorphism was isochemical with except for the addition of H_2_O. Rather than specifying the oxidation state which may be variable, here, the compositions of Oberhänsli et al. (1985) are averaged and simplified to the eight‐component NaO–CaO–K_2_O–FeO–MgO–Al_2_O_3_–SiO_2_–H_2_O (NCKFMASH) model system by neglecting minor MnO, Fe_2_O_3_, and TiO_2_. CaO was corrected for apatite.

The chosen bulk composition consists of the calculated granitic assemblage of biotite (∼12 vol.%), K‐feldspar (∼13 vol.%), plagioclase (∼38 vol.%), and quartz (∼37 vol.%), consistent with reports for Monte Mucrone (Biino & Compagnoni, 1992; Oberhänsli et al., 1985), and the other investigated metagranitoids (Bruno et al., 2001; Bruno & Rubbo, 2006; Compagnoni & Maffeo, 1973; Ibarguchi, 1995; Rubbo et al., 1999; Tropper & Essene, 1999). Because biotite is the only FeO–MgO–H_2_O‐bearing mineral, its modal amount solely depends on bulk composition and remains constant and independent of *P*–*T*. Biotite stores ∼1.7 mol.% H_2_O in the modelled composition, highlighted with the black isocompositional line on Figure 7a (closed system; path A), corresponding to complete H_2_O saturation of the protolith (top scale on Figure 7a). A higher *M*(H_2_O) implies open‐system conditions and the addition of exogenous H_2_O. Full granite H_2_O saturation allows for free H_2_O to be stable only at very low pressure (<2 kbar; indicated by the dotted shading on Figure 7a). The *M*(H_2_O) required for saturation increases with pressure because of the increasing abundance of phengitic white mica and minor zoisite that sequester up to ∼5 mol. % H_2_O at high pressure, corresponding to about 300% of granite H_2_O saturation. At the highest pressure considered here, additional lawsonite is predicted, consuming H_2_O and inhibiting saturation in the investigated *P*–*M*(H_2_O) space (Figure 7a).

Assuming isothermal burial of the fully saturated granite at 600°C (path A on Figure 7a), biotite, two feldspars and quartz with negligible aluminosilicate and muscovite are calculated to about 5 kbar (‘granite’ on Figure 7b). Garnet is predicted from this pressure onward, and plagioclase/albite‐bearing assemblages give way to a diagnostic eclogite facies paragenesis of garnet–Na‐clinopyroxene–phengite–kyanite–K‐feldspar–quartz above around 15 kbar (Figure 7b). K‐feldspar persists due to the low *M*(H_2_O) and would be replaced by phengite ± kyanite given sufficient external hydration (Figure 7a). Contours overlain on the diagram show that *μ*H_2_O increases with *M*(H_2_O) (Figure 7a) and with pressure if composition remains constant (Figure 7b). This trend corresponds to the phase relations shown in Figure 3, but the absolute values of *μ*H_2_O are different due to the temperatures at which Figures 3 and 7 are calculated. Regardless of *P*–*T*, because *M*(H_2_O) and *μ*H_2_O form a pair of extensive and intensive conjugate variables (Powell et al., 2005), the numbers on Figure 7 are independent of bulk composition, and their trend is valid for high‐pressure metagranitoids *s*.*l*. As such, Figure 3 in conjunction with Figure 7 demonstrate that plagioclase breakdown and the related textures form at closed‐system conditions, and unlike in mafic eclogites, this incipient ‘eclogitization’ of metagranitoids does not require exogenous fluid.

At closed‐system conditions and the chosen temperature, H_2_O saturation is only possible below ∼2 kbar (Figure 7) or ≤8 km depth. It is likely that following prograde metamorphism and incipient texture formation, the rocks exhume and cool, further hampering reaction and dehydration. Such a scenario, in the absence of deformation, would allow for preservation of the observed reaction textures. If, however, prograde fluid infiltration occurs, H_2_O is stored in white mica and potentially zoisite (Figure 7a). During subsequent exhumation and decompression the *M*(H_2_O) required for saturation decreases, causing the rock to eventually reach H_2_O saturation (bold line and dotted shading on Figure 7a), leading to more or less extensive dehydration. At fluid‐present conditions, lower pressure retrogression and loss of former high‐pressure assemblages is likely (path B on Figure 7a; Heinrich, 1982; Proyer, 2003; Schorn, 2018), such as the formation of wide‐spread plagioclase–biotite ‘salt and pepper’ textures replacing high‐pressure parageneses in orthogneisses dominating the (U)HP Western Gneiss Region (Hacker et al., 2010).

On the other hand, Young and Kylander‐Clark (2015) suggest that relic plagioclase–K‐feldspar ± biotite partly persisted through burial and UHP conditions in the WGR ortho‐ and quartzofeldspathic gneisses. Only subordinate epidote‐group minerals, phengite, garnet, and rutile locally indicate prograde high‐pressure overprint, but the gneisses lack sodic pyroxene or its retrograde breakdown products. The authors concluded that equilibration has ceased at ‘high‐pressure amphibolite facies’ conditions due to prograde fluid absence. This is consistent with the modelling in Figure 7a: Assuming H_2_O saturation at ∼9–10 kbar (*M*(H_2_O) ≈ 3–4 mol.%; white star on Figure 7a), the rock is expected to consist of biotite–plagioclase–K‐feldspar–muscovite–zoisite–quartz, similar to the dominant orthogneiss‐assemblage of Young and Kylander‐Clark (2015). Further burial would lead to H_2_O absence (Figure 7a) and impede rock‐scale prograde equilibration, causing the partial preservation of igneous assemblages (Young & Kylander‐Clark, 2015). It is expected that relic feldspars undergo partial reaction as described here (path C on Figure 7a), involving albite or jadeite depending on pressure. However, due to the higher *M*>(H_2_O), subsequent exhumation would lead to dehydration at mid‐crustal depth (path B on Figure 7a). The attending retrogression, potentially accompanied by deformation, would cause obliteration of the delicate reaction textures and largely restore ‘high‐pressure amphibolite facies’ assemblages (Young & Kylander‐Clark, 2015).

### 6.3 Implications for high‐pressure metamorphism of granitic crust

The documented reaction textures can develop in orthogneisses s.l. at closed‐system conditions. Due to the paucity of free fluid, reaction rates are likely to be slow, with variable metastable persistence of igneous assemblages in absence of reaction ‘triggers’ such as exogenous fluid infiltration (e.g., Ahrens & Schubert, 1975; Austrheim, 1987) and/or deformation at high pressure (e.g., Gosso et al., 2010; Hobbs & Ord, 2010; Rubie, 1990). Therefore, significant overstepping of solid–solid reactions is required for transformation to occur, with documented ranges between 5 and up to >13 kbar (Austrheim & Griffin, 1985; Wain et al., 2001). However, even though plagioclase breakdown does not strictly require additional H_2_O besides pressure increase to occur (path A on Figure 3), reaction rates are likely enhanced by the addition of H_2_O (path B on Figure 3; Milke et al., 2013; Rubie, 1986) derived from nearby partial biotite breakdown. Assuming a homogeneous mineral distribution in the igneous protolith, plagioclase consumption would occur pervasively because the diffusive length scale of H_2_(O) (∼cm) exceeds the typical grain size, at least in the studied examples where the original plagioclase is entirely lost. Such reaction in favour of denser assemblages promotes the formation of porosity, thereby creating preferential pathways for fluids and/or loci for shear zone development (e.g., Rogowitz & Huet, 2021). If subsequent fluid infiltration and/or deformation occur, progressive equilibration is expected to obliterate the textures, either along the prograde path or due to deformation/dehydration during exhumation (Figure 7a; Proyer, 2003). In fact, the studied rocks preserve reaction textures exclusively in compositionally closed low‐strain domains, whereas nearby shear zones experienced extensive multistage advective fluid infiltration, deformation, and equilibration (Koons et al., 1987; Zucali et al., 2020) with diffusive H_2_O transfer within ∼10 cm from shear zones (Früh‐Green, 1994).

Calculations show that unlike plagioclase‐related reactions (2) and (3), partial breakdown of biotite (reaction 4) may occur at variable *P*–*T* conditions as a function of mineral composition (Figure 2). Coupled biotite and plagioclase breakdown may thus occur at a number of orogenic conditions. It is therefore conceivable that, at the appropriate *P*–*T* conditions, analogous features involving albitic plagioclase instead of jadeite form due to the same mechanisms (Figure 3; Schorn & Diener, 2017). Sodic clinopyroxene is in fact rarely found in (U)HP orthogneisses, despite being widely expected from phase equilibrium calculations (e.g., Palin et al., 2017; Young & Kylander‐Clark, 2015). Instead, in such rocks, the igneous plagioclase typically gives way to albite associated with garnet, phengite, zoisite/epidote, and/or kyanite (Carswell et al., 2000; De Sigoyer et al., 2004; Massonne, 2015; Proyer, 2003; Tursi et al., 2021). This may suggest that prograde incipient equilibration predominantly occurs within the stability field of albite and is followed by its metastable persistence, implying that subsequent equilibration at high(er) pressure is impeded by the lack of additional hydration, deformation, and/or insufficient time scale of metamorphism. Such dry, metastable felsic crust would remain strong and buoyant, as proposed for the Western Gneiss Region (Young & Kylander‐Clark, 2015) and the Tso Morari Massif (Palin et al., 2017).

## 7 CONCLUSION

Unlike in basalt/gabbro or granulite, incipient ‘eclogitization’ of granitoids at high pressure—namely plagioclase breakdown and small‐scale formation of garnet and phengite—does not require exogenous hydration because it can be triggered by H_2_O released during the partial breakdown of igneous biotite. At high pressure, the transfer of biotite‐derived H_2_O into neighbouring plagioclase causes its pervasive replacement by fine‐grained jadeite–zoisite–K‐feldspar–quartz ± kyanite ± phengite. Depending on *P*–*T* conditions, albite occurs instead of jadeite, consistent with the association of albite–garnet–phengite–zoisite/epidote ± kyanite commonly found in metagranitoids and kindred gneisses of many classic (U)HP terranes. Whereas mere pressure increase may suffice to cause plagioclase breakdown, addition of H_2_O from biotite into the texture site likely enhances reaction rates. As such, it can and should occur when granitoids s.l. are subjected to high‐pressure–low‐temperature conditions. Diffusion of FeO–MgO–H_2_O from biotite towards feldspars is accompanied by the formation of complementary coronae involving garnet | phengite–quartz. Minor transfer of CaO from plagioclase to biotite leads to characteristic asymmetric enrichment of grossular in garnet growing towards the plagioclase. A closed system should not hamper the development of reaction textures as summarized here, but both the absence of subsequent hydration and deformation are required to preserve them. Conversely, the reaction textures create structural weaknesses and preferential fluid pathways, thereby favouring subsequent hydration, deformation, and equilibration along the prograde path. In this case, the rocks would dehydrate as they decompress, causing fluid‐assisted retrogression and loss of diagnostic eclogite facies assemblages at lower pressure conditions. The delicate textures described here are therefore only preserved in exceptional cases, even though they may occur in most static closed high‐pressure metamorphic systems.
